# Towards automated joint detection in sleep studies: bridging clinical insight and artificial intelligence

**DOI:** 10.1093/sleep/zsag108

**Published:** 2026-04-20

**Authors:** Alexis Dorier, Pietro-Luca Ratti, Mkael Symmonds, Timothy Quinnell, Gary Dennis, Christine Lo, Michele T Hu, Mauricio Villarroel

**Affiliations:** Institute of Biomedical Engineering, Department of Engineering Science, University of Oxford, Oxford, United Kingdom; Oxford Sleep Centre, Department of Clinical Neurophysiology, Oxford University Hospitals NHS Foundation Trust, Oxford, United Kingdom; Oxford Parkinson’s Disease Centre, Nuffield Department of Clinical Neurosciences, University of Oxford, Oxford, United Kingdom; Oxford Sleep Centre, Department of Clinical Neurophysiology, Oxford University Hospitals NHS Foundation Trust, Oxford, United Kingdom; Respiratory Support and Sleep Centre, Royal Papworth Hospital, Cambridge, United Kingdom; Department of Neurology, Sheffield Teaching Hospitals, Sheffield, United Kingdom; Oxford Sleep Centre, Department of Clinical Neurophysiology, Oxford University Hospitals NHS Foundation Trust, Oxford, United Kingdom; Department of Neurology, Sheffield Teaching Hospitals, Sheffield, United Kingdom; Oxford Parkinson’s Disease Centre, Nuffield Department of Clinical Neurosciences, University of Oxford, Oxford, United Kingdom; Institute of Biomedical Engineering, Department of Engineering Science, University of Oxford, Oxford, United Kingdom; The Podium Institute for Sports Medicine and Technology, University of Oxford, Oxford, United Kingdom

**Keywords:** sleep monitoring, pose estimation, artificial intelligence, computer vision, RBD

## Abstract

**Study objectives:**

To develop and evaluate artificial intelligence methods for detecting upper-body joint positions from video recordings of individuals during sleep, providing a foundation for future automated, video-based analysis of sleep movements that extends beyond conventional sensor-based methods.

**Methods:**

We developed HypnoPose and tested five different model configurations. We pretrained each variant on a public body pose dataset and evaluated on a sleep pose dataset comprising 4419 annotated frames from 198 video segments depicting movement across 74 participants (10 RBD, 4 PD, 9 healthy controls, 51 referred for vPSG screening) recorded in clinical and home settings. We manually annotated each frame with 13 body joints, visibility flags, and head orientation. We evaluated model performance against state-of-the-art pose estimators using precision (mAP) and recall (mAR) metrics based on Object Keypoint Similarity (OKS).

**Results:**

HypnoPose achieved the highest performance (mAP: 0.088, AP@0.5: 0.326) in the sleep domain, doubling baseline HigherHRNet results (mAP: 0.041, AP@0.5: 0.165) and outperforming gold-standard architectures. It showed 40%–120% relative mAP improvement for occluded joints, enhancing detection of the head, shoulders, and elbows. Home recordings showed higher precision than clinic data (mAP 0.14 vs 0.07). Within clinic recordings, NREM stages outperform Wake (mAP 0.11–0.13 vs 0.06).

**Conclusions:**

We present a proof-of-concept for detecting upper-body joint positions from sleep images, even when blankets occlude the person. Our method improves relative precision by 115% compared to standard models. While absolute performance remains modest, this work establishes a first step toward clinically applicable, video-based pose estimation during sleep. Future work should integrate contextual priors and expand annotated sleep datasets.

**Statement of Significance**

Video polysomnography (vPSG) is the gold standard for assessing sleep-related disorders. It is resource-intensive, requires specialist expertise for setup and interpretation, and may not capture typical sleep due to the presence of multiple sensors and the laboratory environment. In some populations, such as individuals with REM sleep behavior disorder (RBD), vPSG can be challenging, and clinically relevant behaviors may not occur during a single recording night. At-home recordings provide greater environmental validity but are often constrained by nonstandard camera positioning and suboptimal lighting. This study introduces a benchmark for automated, frame-wise upper-body pose estimation, to identify anatomical joint positions during sleep. While tools for detecting periodic limb movements and REM sleep without atonia already exist, there remains a critical need for objective, scalable methods to quantify complex movements seen in disorders such as RBD, NREM parasomnias, and sleep-related epilepsies. This work lays the foundation for future automated, noncontact movement analysis that complements existing vPSG workflows, reduces manual review time, and supports accessible monitoring of sleep behavior in both clinical and home environments.

## Graphical Abstract

**Graphical Abstract:**
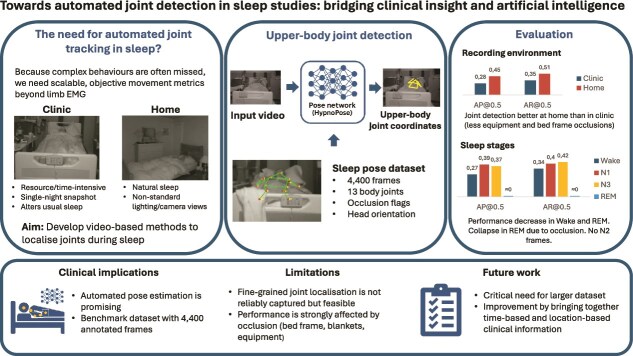

## Introduction

Sleep monitoring through video polysomnography (vPSG) is essential for identifying disorders featuring abnormal movements and behaviors during sleep or upon awakenings, including REM and NREM parasomnias, sleep-related epilepsies, and sleep-related movement disorders. In REM sleep behavior disorder (RBD), the loss of physiological muscle atonia during REM sleep can lead to complex and harmful behaviors such as punching, flailing, or reaching movements [1, 2]. These events often involve prominent upper-limb and emotional facial activity, including vocalizations such as yelling and shouting, that is informative for diagnosis [3–5]. Video polysomnography remains the clinical gold standard for assessing such phenomena, using near-infrared (NIR) cameras to enable recording in complete darkness. However, the complexity of vPSG setup, the need for specialist supervision, and the labor-intensive manual review of video and physiological signals limit its scalability and accessibility in many clinical settings and countries [6–8]. Even for trained clinicians, differentiating RBD from RBD-like conditions such as nocturnal epilepsy or NREM parasomnias can be challenging [1]. Automated analysis of vPSG video using artificial intelligence (AI) offers the potential to assist clinicians by objectively identifying and characterizing motor and behavioral events, reducing review time, supporting diagnostic interpretation, and facilitating large-scale, home-based monitoring [9].

“Body pose estimation” is a computer vision task that identifies and tracks the spatial positions of anatomical joints in the human body, such as shoulders, elbows, wrists, hips, and knees, from images or videos [10]. Using AI [11–19], specifically supervised deep-learning pose estimation models trained on manually annotated images, these body keypoints are detected and connected to form a skeletal representation of the human body. Once extracted, the keypoints can be analyzed to support a wide range of applications, including healthcare [20–22], sports [23–25], and entertainment [26, 27].

Advances in human pose estimation from video cameras using AI have achieved high accuracy in well-lit conditions, where the body is visible without occlusion [19, 28–30]: In 2015, Sun et al. [31] proposed HRNet, a convolutional neural network that preserves high-resolution feature maps throughout the architecture. In 2020, Cheng et al. [18] introduced HigherHRNet, an extension designed for multi-person pose estimation using high-resolution representations. Fang et al. [17] developed AlphaPose, a framework that combines person detection with single-person pose estimation. In 2022, Xu et al. [19] presented ViTPose, a transformer-based model that currently outperforms existing methods by capturing dependencies between joints. Despite their success, these approaches face major technical challenges when applied to sleep studies, where recordings are typically obtained under near-infrared (NIR) illumination. Such conditions produce images with reduced clarity and frequent occlusion from bedding, which can severely reduce model performance.

Previous research in sleep pose analysis has primarily focused on classifying general body postures (e.g. supine, lateral, prone) using wearable sensors, pressure sensors, or video cameras [32–34]. Zhu et al. [33] introduced a method that leverages body-rolling motion and head orientation in videos to detect sleep postures under blankets. Mohammadi et al. [34] applied transfer learning using several pose estimation algorithms to detect posture using a single infrared camera. Tam et al. [35] developed SaccpaNet, an AI model using a depth camera that detects posture by estimating body keypoints. While valuable for providing insights on positional sleep apnoea [36–38] or sleepwalking [35, 39], these approaches are often tested in well-lit environments or lack the granularity to quantify limb movements essential for RBD screening [1]. More recently, Abdelfattah et al. [40] applied optical-flow-based analysis of vPSG videos to automatically identify isolated RBD, underscoring the feasibility of automated video-based movement assessment in clinical sleep recordings. However, such approaches do not capture the spatial configuration of the body or the distribution of limb movements. Despite growing interest in applying computer vision to sleep studies, progress is slowed by the absence of shared, annotated datasets. Collaborative data curation is therefore a necessary step toward clinically reliable video-based movement analysis. To date, no work has specifically addressed body keypoint estimation during sleep while accounting for the combined difficulties of bedding occlusion and very low light levels when there is no ambient illumination.

To address these limitations, we introduce HypnoPose, an AI model for frame-wise upper-body pose estimation from video during sleep in clinic and home environments. HypnoPose establishes upper-body joint localization in individual video frames, which is a prerequisite for future analysis of complex sleep-related movement. We prioritized upper-body pose detection, as previous research has shown that upper-body motion is more discriminative than lower-body activity for detecting RBD-related behaviors [4, 5].

## Materials and methods

### Clinical study

We created a sleep pose dataset within the framework of a clinical study conducted at the John Radcliffe Hospital (Oxford, UK), Royal Hallamshire Hospital (Sheffield, UK), and Royal Papworth Hospital (Cambridge, UK), in collaboration with the University of Oxford’s Nuffield Department of Clinical Neurosciences. The study was approved by the South Central Oxford Research Ethics Committee (reference number 17/SC/0631), recruited 144 participants (44 females, 65.8 ± 14.4 years), including 84 referred for RBD screening, 30 patients diagnosed with REM sleep behavior disorder (RBD), 12 participants with Parkinson’s disease, and 18 age- and gender-matched healthy controls. The demographics of the participants are described in Table 1. Participants underwent vPSG recordings in both sleep laboratory and home environments, the latter to reflect sleep in real-life conditions. Sleep stages for the in-clinic vPSG recordings were manually scored by trained sleep clinicians according to standard criteria.

**Table 1:** Demographics of the participants recruited in the SleepWearables study

Participant group	Count	Gender (M/F)	Age (mean years ± SD)
RBD screening	84	61 M/23F	50.6 ± 20.9
PD	12	7 M/5F	66.0 ± 7,6
RBD diagnosed	30	25 M/5F	68.6 ± 13.1
Healthy controls	18	10 M/8F	72.5 ± 12.6
Total	144	103 M/41F	65.8 ± 14,4

Demographics of the participants recruited in the SleepWearables study. RBD: REM sleep behavior disorder, PD: Parkinson’s disease, M, male; F, female; SD, standard deviation.

**Figure 1:**
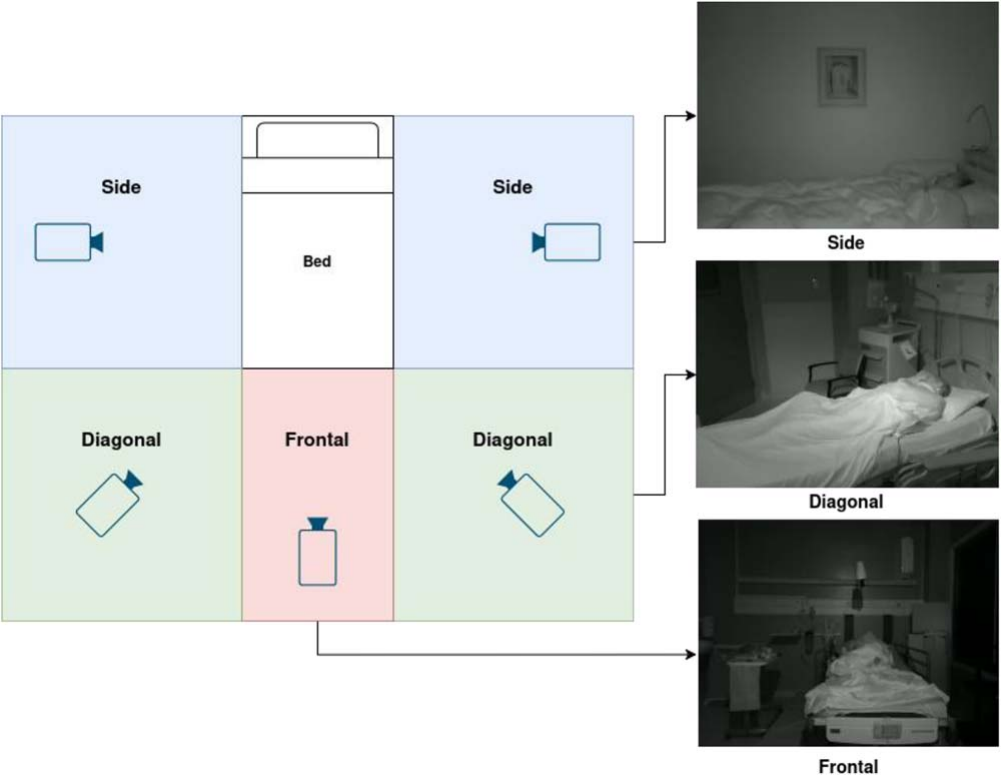
Schematic representation of camera placement angles used during the study. The diagram illustrates the three main camera positions relative to the bed: frontal, side, and diagonal.

Videos from home-vPSG were captured using an NIR camera (SOMNOmedics LAN Infrared Video Camera, 800 × 600 pixels, 25 fps) to enable monitoring in complete darkness without disturbing sleep. The resulting video frames were grayscale intensity images acquired from a single fixed camera. The camera was mounted at a height of 1.5 m and positioned 1.5–3 m from the bed. Camera placement was standardized in clinics but varied in home settings according to room layout (Figure 1). One hundred sixty-six video recordings were collected, totaling 1906 hours (average 11.6 h ± 3.6 h per recording). After quality control to remove videos with poor framing or depicting multiple people, 107 recordings were retained, capturing a variety of camera angles (frontal, diagonal, side) and framing quality (full body, cropped feet, cropped legs).

### Sleep pose dataset

**Figure 2:**
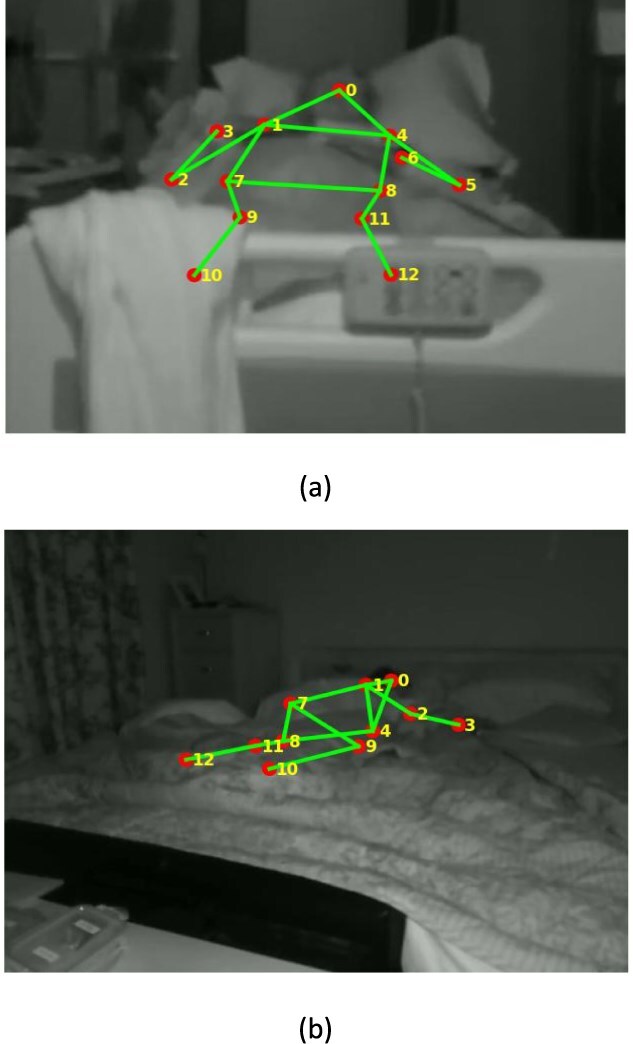
Illustrations of the keypoint annotation process on the acquired grayscale frames: (a) example of annotations in the clinic environment from a frontal view and (b) example of annotations in the home environment in a side view. Dots depict the 13 human body keypoints: (0) head, (1) right shoulder, (2) right elbow, (3) right wrist, (4) left shoulder, (5) left elbow, (6) left wrist (7) right hip, (8) left hip, (9) right knee, (10) right ankle, (11) left knee, and (12) left ankle. The skeleton is shown with bars connecting the keypoints.

We applied a background subtraction [41] algorithm on the sleep videos to identify periods of movement during sleep (see Appendix 1, Figure S1). The algorithms operated on the grayscale frames to generate binary foreground masks used for the motor events identification. The algorithm is characterized by two parameters: the retained number of frames (see Appendix 1, Figure S2) and the minimal pixel intensity difference between frames to flag a background pixel as part of the foreground (see Appendix 1, Figure S3). We tuned these parameters to detect movements as small as hand motions and used morphological operations (erosion and dilation, see Appendix 1, Figure S4) to reduce noise in the resulting binary masks (see Appendix 1, Figure S5). We defined motor events following the guidelines of the International RBD Study Group as periods of motion involving any type of body movement that lasted 0.5 seconds or more and were separated from subsequent events by a minimum interval of 3 seconds [1]. We used this definition to identify and segment periods of sustained movement for annotation and evaluation, as it is not restricted to RBD-specific behaviors. Consequently, brief and isolated motions were not included in the dataset. As motor-based sleep disorders like RBD involve ample and complex motion, we set a minimum threshold for movement, which corresponds to the average size of a participant’s hand. We selected 200 motor events throughout the video recordings using stratified sampling to ensure representation across recording environments, camera angles, and movement amplitudes and durations. We manually annotated the selected motor event using the LabelMe software [42]. We labeled video frames with 13 upper-body keypoints: head, left/right shoulder, left/right elbow, left/right wrist, left/right hip, left/right knee, and left/right ankle (Figure 2) following the COCO format [43]. We merged facial landmarks into a single “head” keypoint. Visibility flags (“visible” or “occluded”) and head orientation (left, right, front, and back) were also annotated. In addition, we annotated bounding boxes enclosing the upper body for each frame. This resulted in 4419 annotated frames from 74 participants. The distribution of the sleep pose dataset reflects real-world sleep scenarios, including both clinical (55%) and home (45%) recordings, with a range of camera angles (frontal, side, diagonal) and movement durations.

### Network architecture

HypnoPose is based on the HigherHRNet [18] architecture (see Appendix 2, Figure S6), a state-of-the-art pose estimation model. This AI model detects body keypoints by analyzing a grayscale image at multiple spatial resolutions simultaneously; higher-resolution features capture fine details, while lower-resolution features provide global pose information. These features are then fused to improve overall performance. To address bedding occlusion and the lack of ambient lighting in the overnight sleep video, we integrated Convolutional Block Attention Modules (CBAMs, (see Appendix 3, Figure S7a)) [44] within the HigherHRNet backbone (see Appendix 3). CBAMs help the model focus on the most relevant visual information by using two mechanisms: channel attention (see Appendix 3, Figure S7b), which emphasizes useful image characteristics such as edges or textures, and spatial attention (see Appendix 3, Figure S7c), which directs focus to important regions in the image, for example, the bed area where the body is located. This allows the network to focus on the body parts despite the challenging visual conditions.

**Figure 3:**
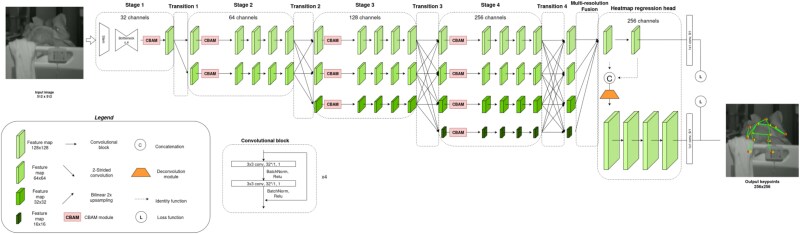
Architecture of the HypnoPose model used for upper-body pose estimation during sleep. Convolutional Block Attention Modules (CBAMs) are inserted at various stages of the network to investigate the impact of attention placement on pose detection performance in sleep conditions. The model follows the HigherHRNet design with multi-resolution parallel branches and fusion across stages. CBAMs are placed either after the stem, within the high-resolution pathway, or at the end of selected stages. The final heatmap output is generated through a combination of multi-scale features.

We evaluated five configurations of HypnoPose, integrating CBAMs into different locations of HigherHRNet (Figure 3), allowing testing of whether attention works best when applied to early fine-detail features, later high-level features, or at multiple points across the network. Specifically, Stage 1 integration targets fine detail at the beginning of processing. Stages 2 and 3 refine the exchange of information between both local and global levels of detail. Stage 4 focuses on global features before the final keypoint predictions. We also tested inserting CBAM at all stages simultaneously to evaluate their cumulative effect. This ablation study allowed us to identify the most effective use of attention mechanisms for pose estimation in sleep recordings. Finally, we adapted the network to predict nine upper-body keypoints (head, shoulders, elbows, wrists, and hips) using 256 × 256-pixel heatmaps.

### Network training

We employed a transfer learning approach to train the different configurations of HypnoPose. We initially pretrained the models on the COCO Keypoints dataset [43] consisting of more than 200 000 images. We adapted this dataset to match the sleep pose dataset by retaining only nine upper-body keypoints (with facial landmarks combined into a single “head” keypoint) and by modifying the bounding boxes to enclose only the upper body. We pretrained the models for 50 epochs using a batch size of 32 images. We used the Adam optimizer with a learning rate of 0.001 and a momentum of 0.9. This pretraining stage provided the network with general pose estimation knowledge before fine-tuning it on the sleep pose dataset.

We applied data augmentation consisting of random rotations (±30°, in 5° increments), scaling (0.75–1.5), and horizontal flipping to simulate variability in body orientation and camera viewpoint without obscuring annotated keypoints. Following pretraining, models were fine-tuned for 50 epochs on the sleep pose dataset. The dataset was divided once into training (80%) and validation (20%) subsets at the recording level, ensuring that all recordings from a given participant were assigned entirely to either the training or validation set to prevent data leakage across splits. The split was stratified to preserve proportional representation of recording environments (clinic and home) and camera angles (frontal, diagonal, side) in both subsets. Because the dataset originates from continuous video, participants contributed unequal numbers of frames depending on recording length. To reduce over-representation of participants with longer videos and mitigate overfitting, we standardized the training data to 60 grayscale frames per participant by downsampling longer sequences and applying the same data augmentation strategy as used during pretraining to increase intrasubject variability where required. As a consequence of this sampling strategy, the dataset does not retain temporal contiguity and is intended for single-image, frame-wise pose estimation rather than sequence-based analysis.

To assess the robustness of model performance to participant sampling variability, we performed a subject-wise fivefold cross-validation analysis for the final selected model (Hypnopose Stage 4). Participants were partitioned into five nonoverlapping folds at the participant level, so that each participant appeared in no more than onefold. We stratified fold assignment to maintain comparable distributions of recording environment and camera viewpoint across folds. For each fold, we fine-tuned the COCO-pretrained model using the same architecture, hyperparameters, data augmentation strategy, and training schedules as in the primary experiment. Then, we evaluated the model on the held-out fold using the same joint localization metrics. We reported the cross-validation metrics as the mean and standard deviation across folds to quantify sensitivity to sampling variability, while the original 80/20 split was used for the ablation study to ensure comparisons across model variants.

We used the mean squared error loss computed between predicted and ground-truth heatmaps for each keypoint (see Appendix 4). Weight initialization was performed using a normal distribution with a mean of 0 and a variance of 0.001 to ensure training stability.

### Evaluation

**Figure 4:**
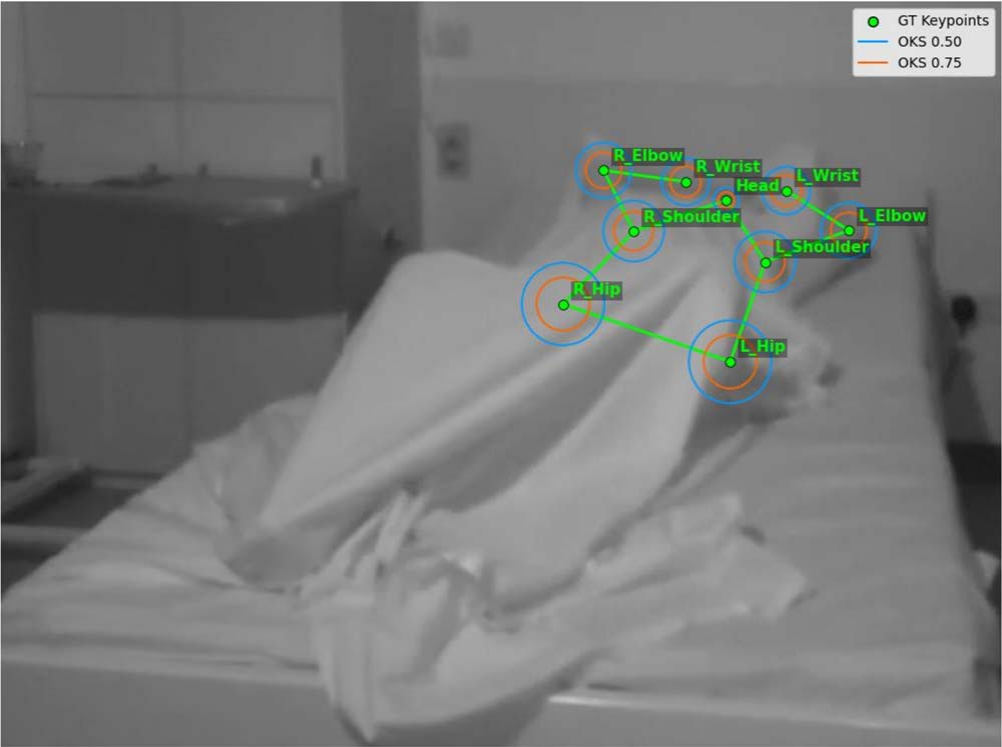
Example of Object Keypoint Similarity (OKS) thresholds used to evaluate detection accuracy. Each predicted joint is compared with its ground-truth position (GT, green) using radial tolerances corresponding to OKS = 0.50 (blue) and OKS = 0.75 (orange). Predictions within these radii are counted as correct detections when computing precision and recall. OKS = 0.50 reflects coarse localization, OKS = 0.75 represents higher-precision joint alignment. The mathematical formulation of OKS is detailed in Appendix 5.

We evaluated model performance using Object Keypoint Similarity (OKS), average precision (AP), and average recall (AR), which are standard in pose estimation research. OKS measures how accurately predicted keypoints align with ground truth. Figure 4 illustrates how OKS is used to compute average precision (AP) and average recall (AR) in our evaluation. Each predicted joint is compared with its corresponding ground-truth annotation using a radial tolerance that scales with the expected localization accuracy for that keypoint. The OKS threshold determines whether a predicted joint is considered correctly localized: an OKS = 0.50 corresponds to a relatively loose matching radius, whereas OKS = 0.75 requires much higher spatial precision. Predictions falling within these zones contribute to the precision and recall values aggregated across thresholds to compute the AP and mean AR. A more detailed mathematical formulation of the OKS metric is provided in Appendix 5.

AP reflects how well the model detects keypoints across varying confidence thresholds, while AR measures how many keypoints are correctly recovered. Mean average precision (mAP) is the average of AP scores across all keypoints and detection thresholds, with AP@0.5 referring to performance at an OKS threshold of 0.5 (see Appendix 5).

These metrics enable the comparison with state-of-the-art baseline models, including HigherHRNet [18], ViTPose [19], and AlphaPose [17], which were trained under similar conditions. Training and validation loss curves were also tracked for each HypnoPose variant to monitor convergence and overfitting.

To gain additional insight into model performance, we computed AP and AR for each keypoint individually.

To assess model performance under different recording and physiological conditions, we selected the best-performing HypnoPose variant and evaluated it separately across recording environments (clinic vs home) and sleep stages, using the subset of clinic recordings for which polysomnography-derived stage labels (Wake, N1, N2, N3, REM) were available. The same COCO keypoint metrics were computed for each subset to assess how lighting, camera placement, and physiological state influenced detection performance.

## Results

Overall, the proposed HypnoPose with attention mechanisms outperformed all baseline models, achieving more than twice the detection precision of the original HigherHRNet.

The sleep pose dataset included 4419 annotated frames from 198 movement video sequences across 89 recordings from 74 participants. The demographics of the participants are described in Table 2. The stratified 80%–20% data split resulted in training and validation sets comprising 59 and 15 participants, respectively. These included 161 motor events and 3500 frames in the training set, and 37 motor events and 919 frames in the validation set. Following data augmentation, the sets were expanded to 3600 and 840 frames, respectively. The train and validation sets are described in Table 3.

**Table 2:** Demographics of the participants included in the sleep pose dataset

Group	Count	Age (mean years ± SD)	Gender (M/F)	Number of recordings	Environments (clinic/home)
RBD screening	51	50.7 ± 21.7	34 M/17F	51	51/0
PD	4	69.0 ± 15.0	2 M/2F	6	0/6
RBD diagnosed	10	70.1 ± 6.5	9 M/1F	20	0/20
Healthy controls	9	75.1 ± 9.6	5 M/4F	12	4/8
Total	74	57.3 ± 21.1	50 M/24F	89	55/34

Demographics of the participants included in the sleep pose dataset. RBD, REM sleep behavior disorder; PD, Parkinson’s disease; M, male; F, female; SD, standard deviation.

**Table 3:** Summary of the train and validation sets of the sleep pose dataset

	Number of recordings					Number of images
	Recording environment		Recording angle			
Data fold	Clinic	Home	Frontal	Diagonal	Side	
Train	88	73	83	62	16	3600
Validation	20	17	21	9	7	840
Total	108	90	104	71	23	4440

Description of the number of recordings in each recording environment, recording angle, and the resulting number of images per fold. “Frontal,” “Diagonal,” and “Side” denote the placement of the camera relative to the bed.

**Figure 5:**
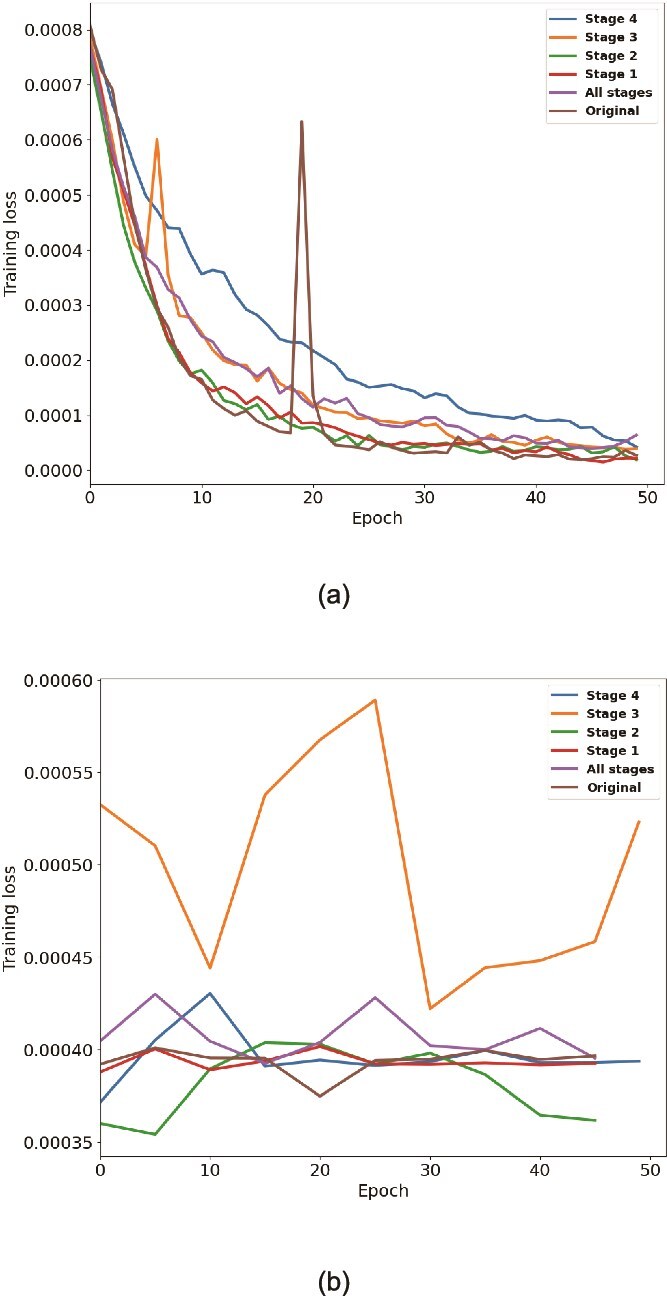
(a) Training and (b) validation losses throughout the 50 fine-tuning epochs on the Sleep Wearables dataset. “Original” displays the losses of the HigherHRNet model, “Stage 1” to “Stage 4” correspond to CBAM variants of the HypnoPose, “All stages” include CBAM in stages 1–4.

Training and validation loss curves over the 50-epoch fine-tuning phase are presented in Figure 5. Training loss consistently decreased across all model configurations, converging by epoch 50. Validation loss curves (Figure 5b) showed a lower variability throughout the training process. Among all the HypnoPose models, the CBAM at Stage 2 variant consistently maintained the lowest validation loss in the latter training epochs.

**Figure 6:**
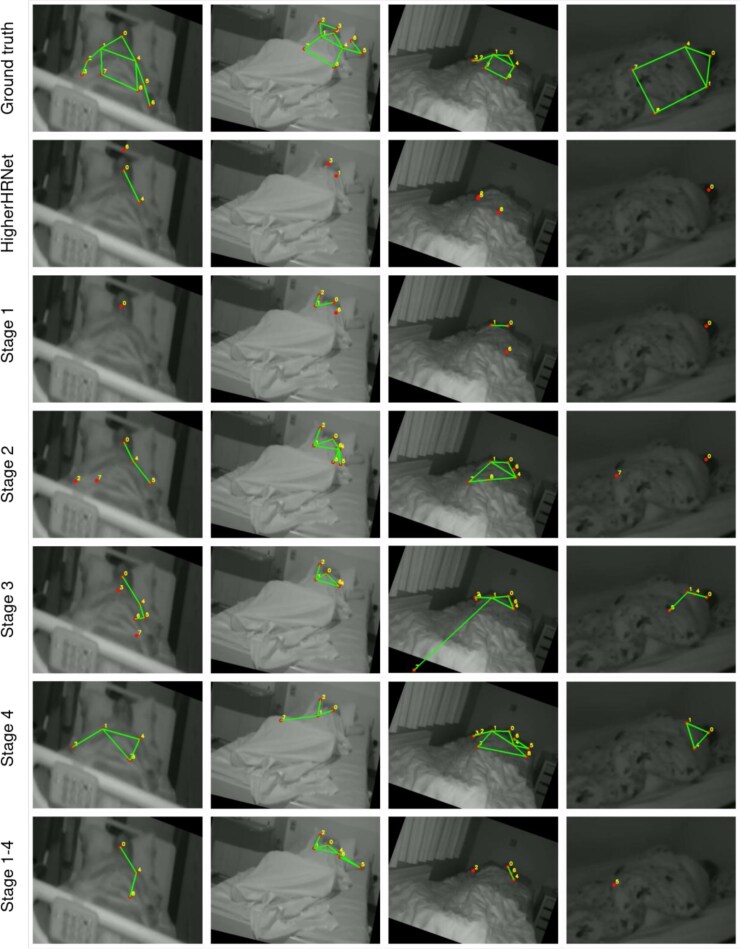
Examples of upper body pose detection performances above a confidence threshold of 0.1. “Ground truth” and “HigherHRNet” correspond to the annotations and the original model, respectively. “Stages” 1–4 are the localizations of CBAMS at the corresponding stages. “Stages” 1–4 correspond to CBAMs included in all stages.

Per-keypoint mean Average Precision (mAP) and mean Average Recall (mAR) are summarized in Table 4. CBAM variants outperformed the baseline HigherHRNet model across most keypoints. Global pose estimation results are presented in Table 5, comparing all models on standard benchmarks. The CBAM Stage 4 variant achieved the highest performance (mAP: 0.088; AP@0.5: 0.326; mAR: 0.131), followed by Stage 2 CBAM (mAP: 0.074). Intermediate results were observed for Stage 1 and all-stage configurations. Both ViTPose and AlphaPose underperformed relative to the CBAM-enhanced models, with mAP scores of 0.067 and 0.036, respectively. Figure 6 displays examples of keypoint detection results across different CBAM configurations and recording environments.

**Table 4:** Per-keypoint evaluation metrics of the HypnoPose variants and HigherHRNet on the sleep pose dataset

Model		Head [0]		R shoulder [1]		R elbow [2]		R wrist [3]		L shoulder [4]		L elbow [5]		L wrist [6]		R hip [7]		L hip [8]	
		mAP	mAR	mAP	mAR	mAP	mAR	mAP	mAR	mAP	mAR	mAP	mAR	mAP	mAR	mAP	mAR	mAP	mAR
HypnoPose	Stage 1	**0.312**	**0.527**	0.259	0.488	0.166	0.388	0.111	0.329	0.260	0.473	0.143	0.370	0.086	0.306	0.046	0.245	0.057	0.260
	Stage 2	0.290	0.511	0.281	**0.535**	0.138	0.347	**0.144**	**0.427**	**0.356**	**0.589**	0.120	0.365	0.071	0.290	**0.110**	0.366	**0.088**	0.331
	Stage 3	0.264	0.502	0.242	0.478	0.155	0.403	0.103	0.338	0.236	0.480	0.152	0.400	0.088	0.311	0.058	0.274	0.033	0.212
	Stage 4	0.285	0.513	0.306	0.529	**0.222**	**0.490**	0.109	0.367	0.337	0.540	**0.197**	**0.436**	**0.095**	**0.342**	0.099	**0.389**	0.083	**0.347**
	Stages 1–4	0.287	0.482	**0.321**	0.515	0.180	0.399	0.110	0.341	0.348	0.542	0.122	0.360	0.083	0.301	0.061	0.310	0.073	0.283
HigherHRNet		0.212	0.422	0.219	0.443	0.152	0.398	0.076	0.305	0.220	0.449	0.121	0.362	0.078	0.287	0.057	0.274	0.070	0.291

“Stage 1” to “Stage 4” denote the CBAM locations within the HypnoPose. “Stages 1–4” denote CBAMs at all stages of our model. mAP = mean average precision, mAR = mean average recall. “L” and “R” denote the left and right body keypoints, respectively. Numbers in brackets display the keypoints indexes. Numbers in bold font represent the top-scoring model for each keypoint.

**Table 5:** Evaluation metrics and comparison with state-of-the-art models on the sleep pose dataset

Model		mAP	AP@0.5	AP@0.75	mAR	AR@0.5	AR@0.75
HypnoPose	Stage 1	0.062	0.232	0.021	0.092	0.309	0.037
	Stage 2	0.074	0.278	**0.023**	0.107	0.355	0.044
	Stage 3	0.055	0.194	0.016	0.088	0.274	0.035
	Stage 4	**0.090**	**0.335**	0.015	**0.136**	**0.406**	**0.057**
	Stage 1–4	0.069	0.271	0.019	0.103	0.329	0.043
HigherHRNet		0.041	0.165	0.009	0.081	0.268	0.031
AlphaPose		0.037	0.227	0	0.074	0.339	0.005
ViTPose		0.076	0.264	0.015	0.13	0.366	0.060

“Stage 1” to “Stage 4” denote the CBAM locations within the HypnoPose. “Stages 1–4” denote CBAMs at all stages of our model. Numbers in bold font represent the top-scoring models. mAP/AR = mean average precision/recall. AP@0.5/0.75 = Average Precision/Recall at Object Keypoint Similarity thresholds of 0.5 and 0.75, respectively.

Table 6 displays the performance of the subject-wise fivefold cross-validation for the HypnoPose Stage 4 variant. Fold-to-fold variability was proportionally modest for AP@0.5 and AR@0.5 but larger for AP@0.75 and AR@0.75.

**Table 6:** Performance of HypnoPose Stage 4 under subject-wise fivefold cross-validation

**Metric**	**Mean ± SD**
mAP	0.120 ± 0.031
AP@0.5	0.350 ± 0.032
AP@0.75	0.061 ± 0.041
mAR	0.159 ± 0.024
AR@0.5	0.415 ± 0.021
AR@0.75	0.107 ± 0.035

Participant-level splitting was used to avoid data leakage between folds. mAP/AR = mean average precision/recall. AP@0.5/0.75 = Average Precision/Recall at Object Keypoint Similarity thresholds of 0.5 and 0.75, respectively.

The Stage 4 HypnoPose variant on the clinic, home, NREM, and wake subsets is shown in Table 7. Home recordings outperformed clinic recordings (mAP: 0.14 vs 0.07; mAR: 0.19 vs 0.11). Stage-specific analysis within the clinic subset showed comparable performance during Wake and NREM stages (mAP: 0.06–0.13; AP@0.50: 0.27–0.39). Only one annotated motor event occurred during REM sleep, for which detection performance was 0 across all metrics. There was no annotated motor event in N2.

**Table 7:** COCO keypoint evaluation by recording environment and sleep stage using the Stage-4 HypnoPose variant

**Subset**	**Annotated frame count**	**mAP (0.50:0.95)**	**AP@0.50**	**AP@0.75**	**mAR (0.50:0.95)**	**AR@0.50**	**AR@0.75**
Clinic	527	0.070	0.280	0.005	0.108	0.347	0.032
Home	291	0.137	0.447	0.048	0.186	0.512	0.103
N1	67	0.127	0.386	0.007	0.151	0.403	0.045
N3	52	0.106	0.373	0.005	0.135	0.423	0.019
REM	18	0.000	0.000	0.000	0.000	0.000	0.000
Wake	390	0.061	0.269	0.006	0.102	0.344	0.033
All frames	818	0.090	0.335	0.015	0.136	0.406	0.057

Clinic vs home and sleep-stage analyses were computed using the same COCO metrics (mAP, mAR, AP@0.5, AP@0.75, AR@0.5, AR@0.75). The REM subset includes only one annotated motor event and should not be interpreted as representative of general REM performance. There were no annotated frames in N2 sleep. Subgroup analysis is exploratory and not for stage-specific inference

## Discussion

### Clinical context and study aim

Motor-based sleep disorders like RBD present diagnostic and monitoring challenges, with current assessment relying on resource-intensive polysomnography and subjective reporting from patients and bed partners. Automated pose estimation and tracking during sleep could support more objective, continuous monitoring of sleep behaviors, including in home environments. For clinical relevance, a sleep body pose detection method must reliably identify anatomical landmarks relevant to both coarse and subtle movements. In the context of RBD, this includes large-scale motions such as limb flailing or trunk movements and finer gestures such as reaching, waving, or twitching. Accurately detecting body joints in sleep recordings is particularly challenging under the quality of near-infrared imaging, the occlusion by bedding, and the variable camera viewpoints that reduce visibility, which is reflected in the performance across HypnoPose configurations.

### Principal findings

Results show that upper-body joint localization from near-infrared images is feasible but remains limited in precision. Across models, performance supports posture-related localization, whereas the consistently low AP@0.75 indicates that precise localization of distal joints, such as wrists and elbows, is not yet reliable under typical sleep conditions. Recent work [40] has demonstrated that automated video analysis can distinguish isolated RBD from other sleep disorders using vPSG recordings. Our study complements this approach by targeting the anatomical localization of movements through keypoint detection rather than global motion features, enabling future quantitative descriptions of which body parts move and how they move. This distinction is critical for future efforts aiming to characterize complex behaviors such as dream enactment or focal limb jerks, which cannot be resolved from global motion metrics alone.

### Model learning behavior and effect of attention

Training and validation curves help assess whether models are learning stably and how they perform on unseen grayscale frames. The similar convergence of training losses across HypnoPose variants (Figure 5a) indicates that adding attention modules into the HigherHRNet architecture did not alter the ability to fit the training data during fine-tuning on the sleep pose dataset. In contrast, differences in validation loss patterns (Figure 5b) suggest that where attention is inserted can affect performance on unseen data. The variability in the validation loss emphasizes the difficulty of adapting pose models to near-infrared sleep recordings with occlusion and limited visual detail.

Among the HypnoPose variants, the Stage 2 configuration showed the most consistent learning behavior. Although differences were modest, this is a balance between enhancing local image details and preserving larger-scale posture information. In contrast, the instability observed in the Stage 3 variant suggests that attention at this level may reduce performance on unseen data. The persistent gap between training and validation behavior across variants indicates that dataset size and diversity remain limiting factors, supporting the need for larger, sleep-specific annotated datasets.

### Keypoint-level performance and clinical interpretation

A keypoint-level analysis is clinically relevant, as accurate localization of upper-limb joints (e.g. wrists, elbows) is essential for detecting RBD-related behaviors such as punching, flailing, or reaching. Table 4 shows that performance drops when transferring from COCO pretraining to the sleep pose dataset, emphasizing the difficulty of applying general-purpose models to near-infrared recordings. The models pretrained on the RGB dataset rely on color and textures, which are absent in near-infrared images used in sleep studies. The underperformance of the baseline HigherHRNet supports the value of adding attention mechanisms designed to improve feature selection under low-visibility conditions.

Early-stage CBAM integration improved head detection, likely because the head is often the most visible body part during sleep. This suggests that early attention enhances fine visual details required for detecting visible regions. Accurate head localization could enable downstream posture estimation and a more interpretable movement description.

Shoulder detection improved consistently across models, with the all-stages CBAM model achieving the best performance. Clinically, shoulders provide anatomical anchors for inferring posture and upper-limb position. In contrast, elbow and wrist detections were more challenging. While elbows were detected with moderate accuracy, wrists consistently showed the lowest performance across models. From a clinical perspective, this represents a critical limitation: distal joints are often required to characterize smaller and clinically meaningful movements.

Hip localization improved modestly but remained less reliable than upper-body keypoints. The observed performance gradient (head > shoulders > elbows > wrists > hips) reflects the typical bedding occlusion pattern of supine sleep, where lower body regions are frequently covered. This suggests that visible landmarks, such as the head and shoulders, may be the most reliable priors for inferring posture, while distal and occluded keypoints remain difficult to estimate accurately.

### Comparison across models and implications for domain transfer

Table 5 shows that all HypnoPose variants outperformed the baseline HigherHRNet model, supporting the benefit of attention mechanisms in this domain. The Stage 4 CBAM configuration achieved the best overall performance, suggesting that attention at lower resolutions can help capture broader posture context and partially compensate for occlusion. Conversely, the weaker performance of AlphaPose and ViTPose reinforces the limitations of transferring models trained on well-lit, unobstructed datasets to the sleep domain. ViTPose may be limited by the small dataset by not fully exploiting the efficacy of its transformer-based attention mechanisms. The consistently low AP@0.75 across models highlights the challenge of achieving precise keypoint localization under near-infrared settings with limited data. Direct adaptation of existing models optimized for general activity recognition is insufficient. These findings support the need for domain-specific approaches that account for occlusion patterns, visible keypoints, and the low-light visual characteristics of sleep environments.

The subject-wise fivefold cross-validation of the HypnoPose Stage 4 variant (see Table 6) showed consistent performance across splits, supporting that the reported results are not driven by a single favorable train/test partition. However, variability increased at stricter localization thresholds, highlighting that precise joint detection remains unstable.

COCO pretraining may be suboptimal for body pose estimation during sleep, as it does not reflect the context relevant to sleep conditions. Additionally, keypoint detection was not constrained to the bed area (Figure 6, third column). Incorporating a bed-region constraint and anatomical or spatial priors on likely keypoint locations could reduce implausible detections and improve detection robustness.

### Performance by environment and sleep stage

Analyses stratified by environments and sleep stages provide additional context. Detection accuracy was higher in home recordings than in clinic recordings, suggesting that acquisition conditions have a measurable impact on performance. In clinic settings, cameras were often positioned at lower or more horizontal angles, which may have reduced visible body surface area and made joint localization more difficult.

Within the clinic subset, NREM stages (N1 and N3) showed higher detection accuracy than Wake, consistent with reduced motion blur during periods of relative stillness. Lower performance during Wake is consistent with more frequent and rapid motion. Results for REM sleep should be interpreted with caution, as only one annotated motor event was available for this stage. These findings remain exploratory and emphasize the importance of balanced representation across sleep stages when building annotated datasets for model development and evaluation.

The qualitative examples shown in Figure 6 support the results displayed in Table 4. The first three columns of Figure 6 suggest that models perform better on images captured frontally and diagonally, which were more common in the dataset than strict side views. Consistent camera framing a viewpoint facilitates the feature learning and generalization on unseen data. These observations support standardized video acquisition protocols where feasible and motivate training on more diverse viewpoints when the goal is generalization to real-world home settings.

### Limitations, data needs, and clinical relevance

Despite these improvements, current performance remains insufficient for clinical deployment. The model shows potential for coarse posture estimation, but inconsistent localization of distal joints limits the ability to describe small, clinically meaningful movements. Improving clinical utility will require stronger anatomical constraints incorporating temporal consistency across frames.

A major limitation of this study is the size and diversity of the video dataset. Although the videos capture real sleep conditions, the number of annotated frames and the representation of postures and stages are limited, which constrains generalization. High-quality sleep pose annotations are extremely time-consuming, and the medical nature of the data limited the feasibility of additional redundant annotations and inter-rater assessment in this study.

Given the sensitive medical nature of the recordings, sharing and annotating them strict privacy and ethical considerations. This emphasizes the need for collaborative efforts within the research community to establish shared sleep pose datasets, appropriately governed sleep pose datasets that enable benchmarking and reproducibility.

The clinical impact of automated sleep pose estimation will depend on demonstrating that pose-derived outputs can improve or streamline clinical workflows, reduce manual video review burden, and support scalable monitoring. This will require both improved pose detection accuracy and validation of clinically meaningful measures derived from pose trajectories.

Unlike depth-camera approaches that enable 3D skeleton detection [45], our method estimates 2D joint localizations from a single near-infrared camera as used in typical vPSG procedures. In these methods, joint locations are visualized as a skeleton via predefined anatomical connections. We do not enforce explicit skeleton or torso constraints in this study, which contributes to anatomically implausible detections, as shown in Figure 6.

The advances in automated sleep video analysis will depend on community efforts to build larger, standardized, and well-annotated video datasets. The scarcity of accessible sleep video data represents the main challenge to reproducible benchmarking. A multi-institutional repository, with consistent annotation of body keypoints and movement-related labels, under appropriate privacy controls, would enable reliable comparison of methods. The present work provides a baseline that emphasizes the feasibility and clinical importance of this collaborative direction.

## Conclusion

We present a proof-of-concept model that adapts a state-of-the-art pose estimation technique to the specific visual and anatomical challenges of sleep. Future progress will depend less on architectural refinements than on the creation of larger, annotated, and clinically diverse datasets. Collective efforts to share de-identified sleep video recordings and establish common annotation standards would enable reproducible benchmarking and accelerate the translation of computer vision methods into sleep research. In parallel, integrating domain-specific priors, such as bed geometry and head orientation, and modeling temporal dynamics across video sequences could enhance movement tracking. These developments would improve the accuracy, interpretability, and clinical relevance of video-based pose estimation, supporting quantitative, scalable tools for studying motor activity in sleep.

## Supplementary Material

supplementary_materials_zsag108(1)

## Data Availability

The code used in this work, including model implementation and pretrained weights on the motor events dataset, will be made publicly available at https://github.com/Dorale. Due to the medical nature of the underlying dataset, the raw data cannot be shared.

## Notes

Clinical Trial

Study Title: Optimizing wearable technology for remote diagnosis and monitoring of sleep disorders

Internal Reference Number / Short title: Wearable technology for the diagnosis & monitoring of RBD

IRAS Project Number: 218932

REC Reference number: 17/SC/0631

Chief Investigator: Prof. Michele Hu, Oxford

Sponsor: University of Oxford

Funder: Funded by the National Institute for Health Research (NIHR) Oxford Biomedical Research Centre (BRC)
