# Supplementary material for: Towards automated joint detection in sleep studies: bridging clinical insight and artificial intelligence
Source: Sleep. 2026 Apr 20;49(7):zsag108. doi: 10.1093/sleep/zsag108 (PMC13357495; doi:10.1093/sleep/zsag108)
Supplement: supplementary_materials_zsag108(1) [file supplementary_materials_zsag108(1).docx]

**Towards automated joint detection in sleep studies: bridging clinical insight and artificial intelligence**

Alexis Dorier^1^; Pietro-Luca Ratti^2,3^; Mkael Symmonds^2^; Timothy Quinnell^4^; Gary Dennis^5^; Christine Lo^2,5^; Michele T. Hu^3^; Mauricio Villarroel^1^

1. Institute of Biomedical Engineering, Department of Engineering Science, University of Oxford, UK
2. Oxford Sleep Centre, Department of Clinical Neurophysiology, Oxford University Hospitals NHS Foundation Trust, Oxford, UK
3. Oxford Parkinson’s Disease Centre, Nuffield Department of Clinical Neurosciences, University of Oxford, UK
4. Royal Papworth Hospital Respiratory Support and Sleep Centre, Cambridge, UK
5. Department of Neurology, Sheffield Teaching Hospitals, Sheffield, UK

Corresponding author:

Mauricio Villarroel

mauricio.villarroel@eng.ox.ac.uk

Institute of Biomedical Engineering

Old Road Campus Research Building

Headington

Oxford OX3 7DQ

**Supplementary material**

1. **Motor events detection**

**Background and foreground segmentation**

We followed the work proposed by Zivkovic et al.^[[1]](#footnote-1)^ Given a sequence of successive images recorded by a fixed camera, we define a probability density function over time for each pixel separately. We assume that neighbouring pixel values are uncorrelated to each other.

Let $x_{t}$ be the value of a single pixel of a greyscale image at time $t$. To adapt to events such as moving objects or illumination changes, we define the training sequence at time $t$ as $\mathcal{H}t={\{x}_{t-T},\ldots,x_{t}\}$, where $T$ is the history length. The training sequence $\mathcal{H}$ is a moving window that contains $T$ pixel values for the pixel $x$ and is updated each frame by adding a new sample $x_{t+1}$ and discarding the oldest one $x_{t-T}$. As $\mathcal{H}$ contains values that belong to both foreground (FG) and background (BG), we define a corresponding set of indicators $\mathcal{B}t=\{bt-T,\ldots,b_{t}\}$ as:

Equation 1

$$b_{t}=\left\{ \begin{aligned} 0, if x_{t}\in FG \\ 1, if x_{t}\in BG \end{aligned} \right.$$

The first training sequence $\mathcal{H}_{0}$ was initialised using the first $T$ samples of each video recording, all the values in $\mathcal{B}_{0}$ were initialised to one (i.e. all pixels belong to the background). The probability density function of $x_{t}$ given $\mathcal{H}$ is defined by kernel density estimation as:

Equation 2

$$\hat{P}\left( x_{t} | \mathcal{H}_{t} \right)=\frac{1}{TD}\sum_{m=t-T}^{t} \mathcal{K}\left( \frac{\left\| x_{m}-x \right\|}{D} \right)$$

where $D$ is the distance threshold that defines the influence of past pixel values on the probability density function of $x_{t}$. The kernel function $\mathcal{K}$ is defined as:

Equation 3

$$\mathcal{K}\left( u \right)=\left\{ \begin{aligned} \text{1, }\text{if }u<\frac{1}{2}\text{ } \\ 0,\text{ otherwise} \end{aligned} \right.$$

The probability that a pixel value $x_{t}$ belongs to the background given the history $\mathcal{H}$ is given by:

Equation 4

$$\hat{P}\left( x_{t}=BG | \mathcal{H}t \right)=\frac{1}{TD}\sum_{m=t-T}^{t} b_{m}\mathcal{K}\left( \frac{\left\| x_{m}-x \right\|}{D} \right)$$

If equation 4 is greater than a threshold $c_{thr}$; we define $x_{t}$ as being part of the background. The threshold value is given by:

$$c_{thr}=\frac{k}{TD}$$

where $k$ is the number of background samples contained within the distance $D$ needed for equation 4 to influence the classification of $x$ as BG. Following Zivkovic et al.'s methodology, we used $k=\left[ 0.1T \right]$, where $\left[ . \right]$ is the round-to-integer operator.

The background subtraction algorithm can be generalised in three steps applied to each pixel of new video frame independently:

1. Classify the new sample $x_{t}$ using Equation 4. If the value is greater than $c_{thr}$, the new pixel value is considered as part of the background. Otherwise, it is part of the foreground.
2. Update $p\left( x_{t} | \mathcal{H}_{t} \right)$ using equation 2 by adding the next sample $x_{t+1}$ to $\mathcal{H}t$ and discard the oldest datapoint $x_{t-T}$ in the training sequence.
3. Update $p\left( x_{t}=BG | \mathcal{H}_{t} \right)$ in equation 4 by setting the corresponding value of $b_{m}$ of the new sample using equation 1.

If a foreground object stops moving, the number of samples $k$ within distance $D$ will increase the value of equation 4. Equation 4 will eventually become greater than $c_{thr}$, the next sample $x_{t}$ will then be considered as part of the background.

When this method is applied to each pixel of a video recording separately, the algorithm results in binary masks that differentiate pixels that belong to the foreground from the background, as seen in Figure S1. Two parameters need to be defined experimentally to tune the algorithm's sensitivity to motion: the history length $T$ and the pixel distance threshold $D$.


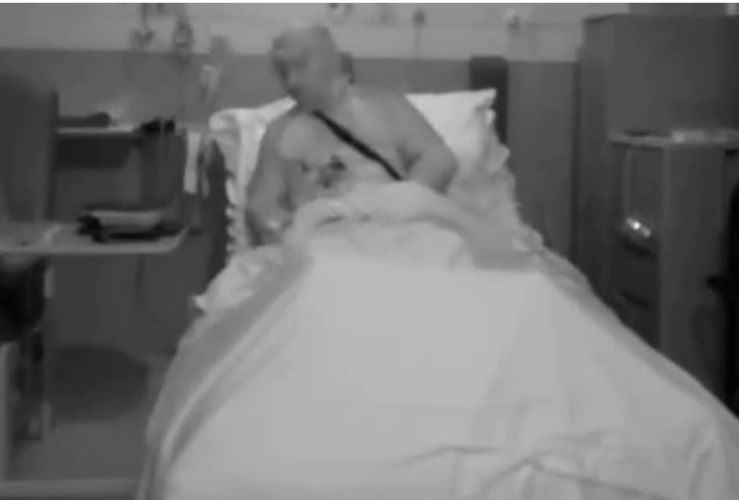

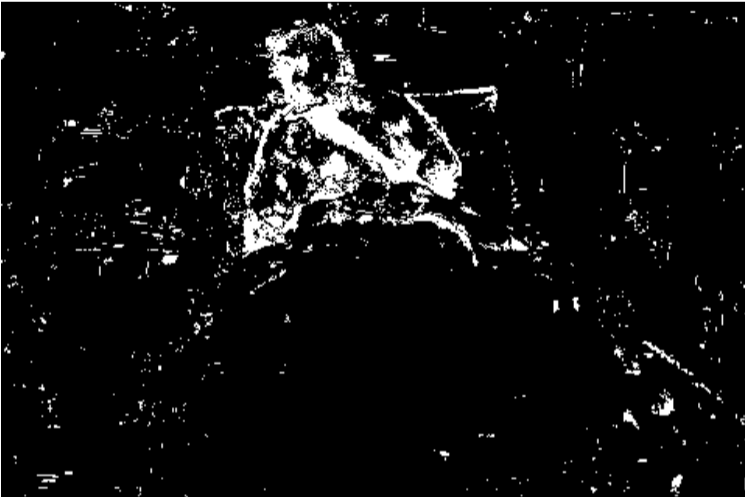


(a) (b)

Figure S1 - Example of foreground segmentation method: (a) Reference frame. (b) Resulting binary. The pixels in white are part of the detected moving foreground

**Parameters tuning**

Patients diagnosed with RBD often move not only multiple large muscle groups such as upper and lower limbs, but also smaller single body parts such as a hand or a foot. Therefore, we defined the movements of a finger as the minimum body movement area to detect in the recorded videos. We manually browsed videos to find multiple segments that depicted a single finger movement and experimentally adjusted the history length $T$ of the background subtraction algorithm. Figure 2 suggests that a larger history length $T$ results in a more reliable detection of the moving foreground areas, while reducing noise. A larger history increases the number of background pixels needed for a new pixel to be reconsidered as part of the background. This is because equation 4 requires more background samples to exceed the threshold $c_{thr}$. Consequently, a pixel remains longer in the foreground masks before blending in the background. Figure S2 suggests that the detected foreground areas using $T=150$ and $T=300$are comparable. We selected a history size of$T=250$ frames as a compromise between movement and noise detected.


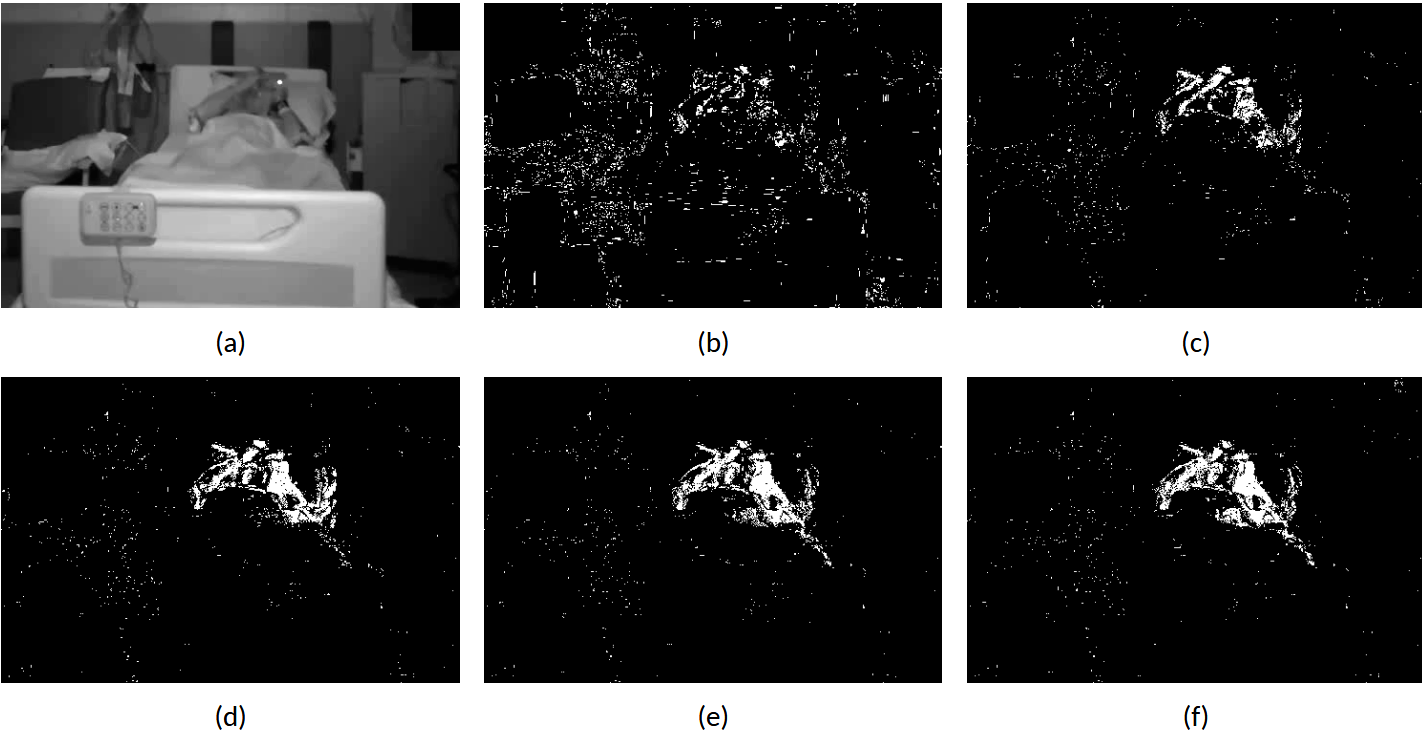
Figure S2 - Example of the influence of the history length T on the foreground masks. (a) The original frame showing an upper limbs movement. (b) to (f) the detected foreground pixels (in white) corresponding to the motor event. History length varied as (b) $T=10$, (c)$T=75$, (d) $T=150$, (e) $T=250$, (f) $T=300$. The distance threshold $D$ is set to $D=10$.

A higher distance threshold $D$ increased the number of neighboring datapoints influencing the probability density function of $x$ (equation 4) and decreased the value of the threshold $c\_thr$ (equation 5), lowering the sensitivity of the algorithm to moving objects. Increasing the value of $D$ reduced the noise but reduced the foreground areas corresponding to body parts movements as seen on Figure S3. Accordingly, we set the distance threshold to $D=10$, for the algorithm to be sensitive enough to detect small amplitude movements.


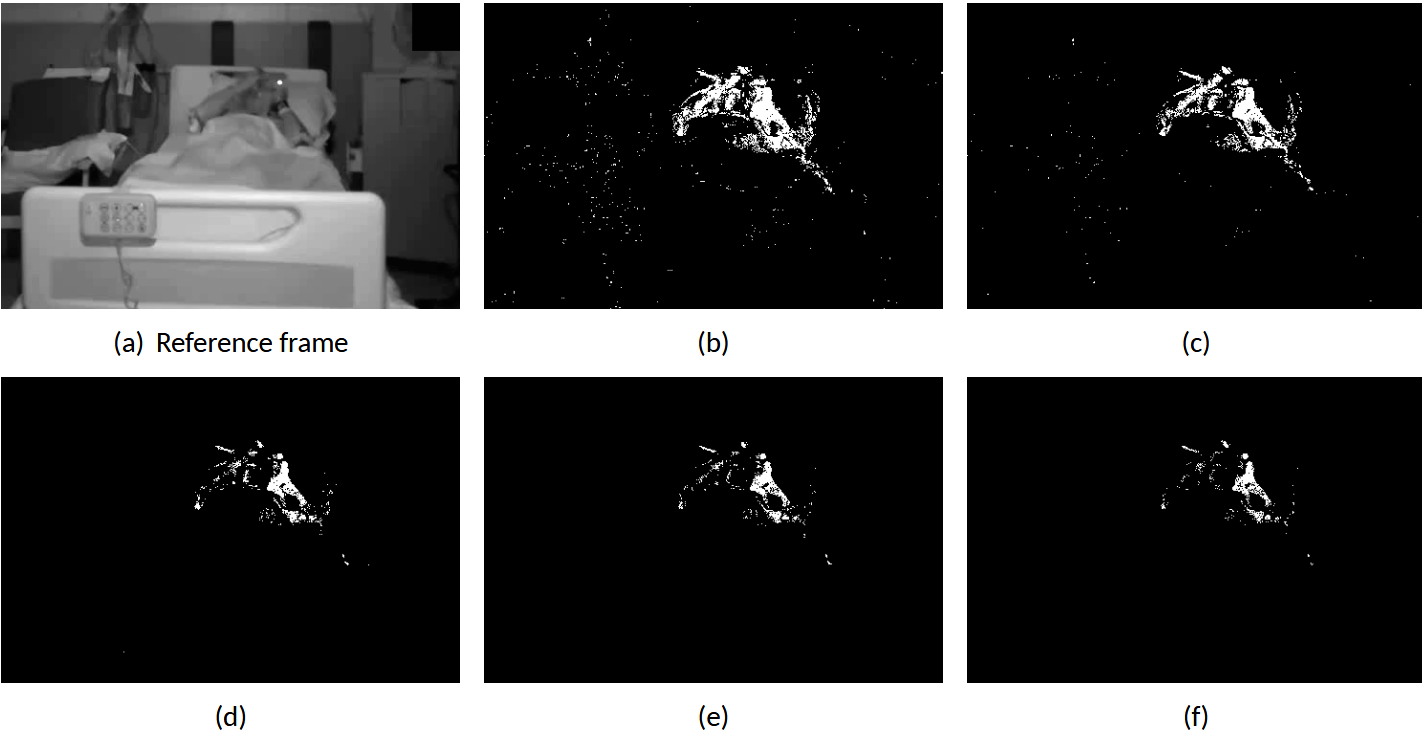
Figure S3 - Example of the influence of the distance threshold $D$ on the foreground masks. (a) Original frame showing upper limbs moving. (b) to (e) The detected foreground pixels (in white) corresponding to the motor event. Distance thresholds varied as (b) $D=10$, (c) $D=25$, (d) $D=50$, (e) $D=75$, (f) $D=100$.

**Foreground mask denoising**

We tuned the distance threshold $D$ and history length $T$ for high sensitivity to detect events such as the movement of fingers of the participants. As a consequence, a significant amount of noise was included as part of the foreground masks. To reduce the noise in the foreground masks, we performed morphological closing and opening operations. The subsequent application of closing and opening operations leave larger regions unaffected, while removing smaller objects and gaps. The shape (Figure S4) and size (Figure S5) of the structuring element influence the morphology of the output binary mask. It is necessary to select these two parameters for the output to effectively discard the background noise while preserving the shape of the foreground areas of interest.


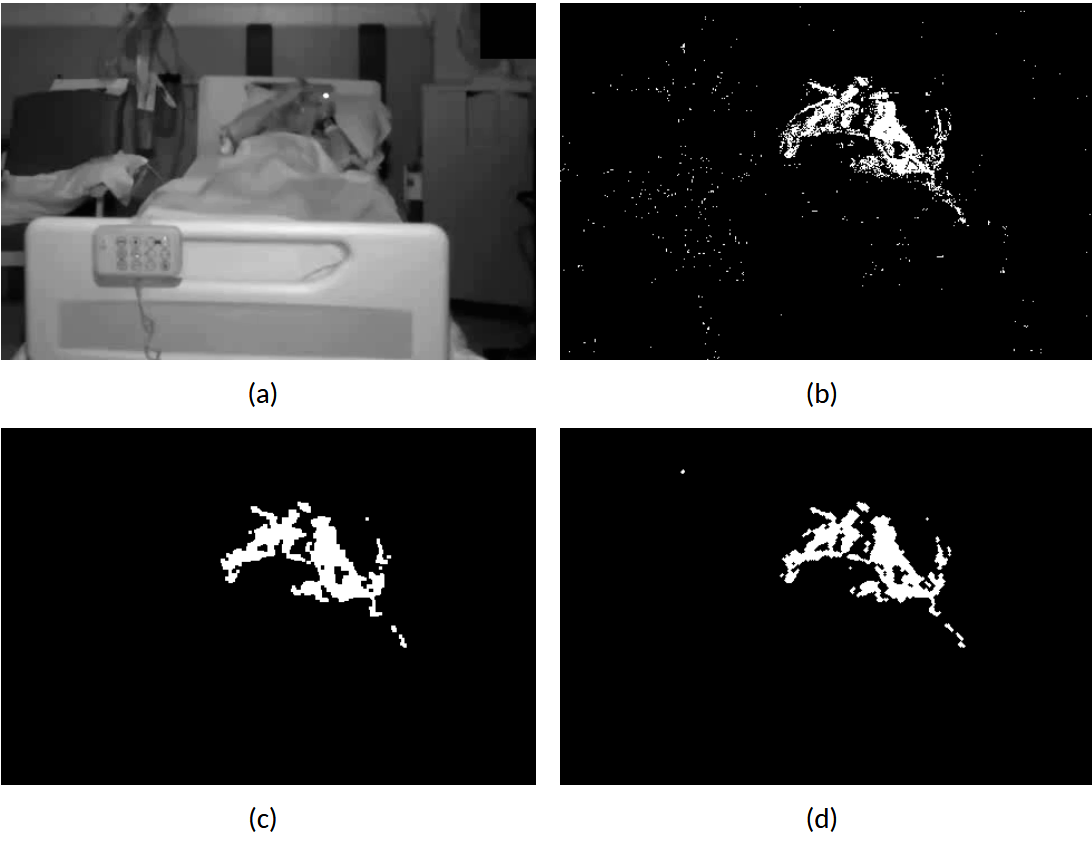


Figure S4 – Influence of the kernel shape on morphological closing and opening on a foreground mask. (a) Original frame showing an upper limb movement. (b) Output of the foreground segmentation algorithm. Binary mask after closing/opening morphological operations with (c) a square kernel of size 3, (d) an elliptical kernel of size 3.

The denoising process should discard noise without affecting the pixels that correspond to the body parts in motion. As shown in figure 4, closing and opening the binary foreground mask with a square-kernel modifies the foreground mask unnaturally, the foreground mask is left pixelated with squared edges. An ellipse-shaped kernel preserves the continuity of the foreground pixels uniformly. Thus, we used an ellipse-shaped kernel for closing and opening the binary masks.


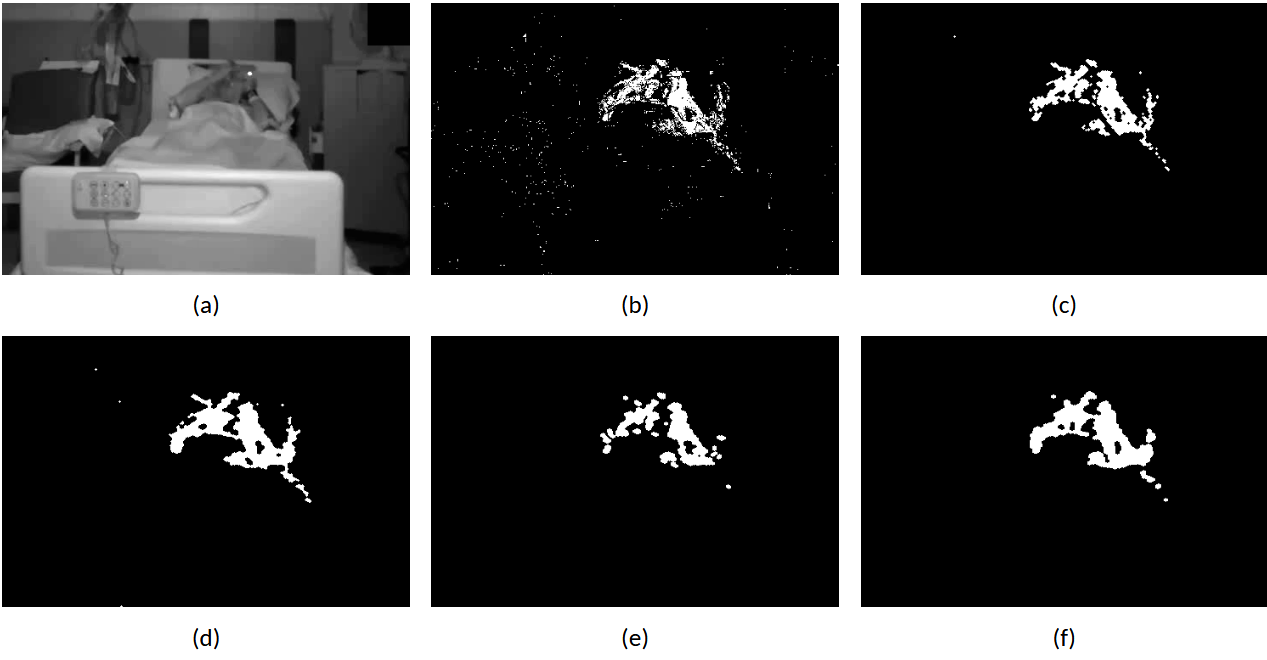
Figure S5 - Influence of the kernel size on morphological closing and opening operations on a foreground mask. (a) Original frame showing an upper limb movement. (b) Output of the foreground mask. (c) to (f) Influence of closing and opening kernel size on the foreground binary mask. Closing (C) and opening (O) kernel sizes varied as (c) C = 3, O = 3 (d) C = 5, O = 3 (e) C = 3, O = 5 (f) C = 5, O = 5

As shown in Figure S5, a large closing kernel size fills the gaps within the foreground mask more effectively. However, the size of the kernel requires a trade-off between discarding the noise and preserving the outline shape of the foreground area. After closing and opening a foreground mask, the detected foreground's integrity should be enhanced by filling the gaps present within the shape, while discarding the unwanted noise. Thus, we selected closing kernel size of $5\times5$ pixels to fill the gaps, and used an opening kernel of size $3\times3$ pixels to discard the noise while preserving the outline of the foreground.

1. **HRNet and HigherHRNet**

High-Resolution Network (HRNet)^21^ differs from conventional architectures by maintaining high-resolution feature representations throughout the network. Traditional deep networks, such as ResNet^31^ and VGGNet^32^, progressively downsample spatial resolutions to extract high-level semantic features. In contrast, HRNet retains high-resolution representations at every stage while incorporating multi-resolution features. This architectural design enables HRNet to capture both fine-grained spatial details and coarse contextual information, making it effective for pose estimation tasks.

HRNet is structured around parallel convolutional streams operating at multiple resolutions, as shown in Figure S6. It maintains four parallel branches; each processing feature maps at different resolutions (Figure S6 legend). Initially, a high-resolution stream called the stem is established, which consists in two 3x3 convolutions with a stride of 2 in the second convolution, reducing the input image resolution from 256x256 to a feature map of 128x128 to double the receptive field and increasingly capture global context. Following this, four bottleneck modules are used for feature learning, each consisting in three consecutive convolutions with kernel sizes of 1x1, 3x3 and 1x1 respectively, separated by a batch normalisation and a ReLU function. Then, four stages undergo multi-resolution features exchanges through transition layers.

Stage 1 is a high-resolution stream (128x128) which is established and refined using a residual “convolutional block” (Figure S6 legend), which consists in two 3x3 convolutions that are each followed by a batch normalisation and a ReLU function. Stage 1 features undergo the first transition: Features of Stage 1 are downsampled using 2-strided convolutions, introducing Stage 2; a second parallel branch with half the resolution (64x64).


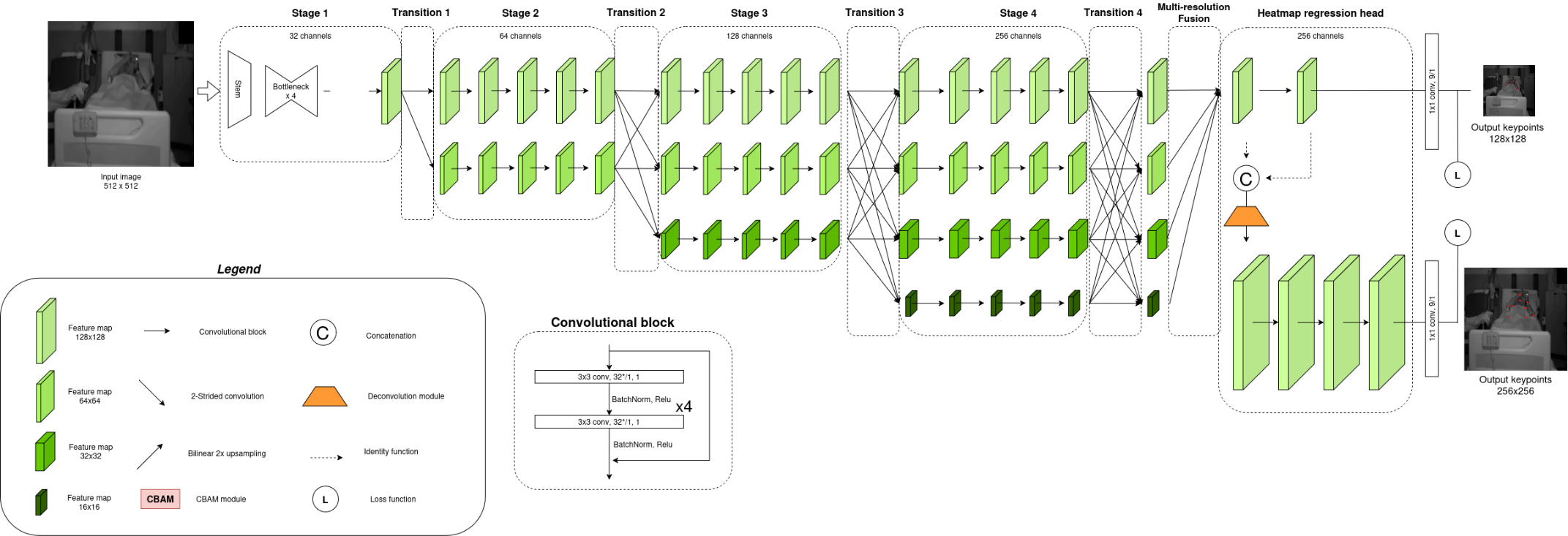


Figure S6 – Overview of the HigherHRNet architecture. The network uses the HRNet^33^ backbone, which maintains high-resolution features throughout the model by continuously exchanging information between parallel streams. HigherHRNet generates the keypoints’ heatmaps at two different scales, which is done by assigning a loss function to each output stream during training^21^.

Each stream of Stage 2 undergoes four convolutional blocks before transition 2: Lower-resolution branches are upsampled using bilinear interpolation, while higher-resolution branches are downsampled via 2-strided 3x3 convolutions before fusion. A new lower resolution stream (32x32) is created to introduce stage 3. After the four convolutional blocks of stage 3, upsampling and downsampling processes are repeated to generate a fourth low-resolution stream (16x16). The four parallel streams of features maps go through 4 convolutional blocks for feature extraction. The four resulting feature maps (128x128, 64x64, 32x32, 16x16) are fused through 2/4/8-strided convolutions and bilinear interpolations with factors of 2/4/8 to obtain a refined feature map for each resolution stream.

Building upon HRNet’s architecture, HigherHRNet^21^ introduces a scale-aware high-resolution network that increases the precision of detection (Figure S6, heatmap regression head), particularly for small keypoints and in multi-person pose estimation.

While HigherHRNet retains the core parallel multi-resolution streams of HRNet shown in Figure S1, it introduces modifications that improve keypoint localisation. HigherHRNet extends HRNet by adding a 1/2 resolution stream using a deconvolution module after the multi-resolution feature fusion black of the HRNet backbone (Figure S6, right side). The deconvolution module consists of three consecutive transposed convolution layers: Each transposed convolution layer uses a kernel size of 4x4, a stride of 2, and a padding of 1, effectively doubling the spatial resolution at each step. Batch normalisation and ReLU activation are applied after each deconvolution to stabilise training and the final output resolution reaches 1/2 of the original input resolution, providing an additional representation of keypoint locations. Each keypoint is then regressed using 1x1 convolutions in both 128x128 and 256x256 resolutions. The 128x128 keypoints heatmaps are upsampled to 256x256 using bilinear interpolation and averaged with the corresponding 256x256 keypoint heatmap. The keypoints are then detected using the highest value of the averaged heatmap.

During training, each output resolution stream (128x128 and 256x256) is assigned specific ground truth heatmaps corresponding to its spatial scale. This is done by generating the target keypoints’ heatmaps for the two resolutions represented as 2D Gaussian distributions centred on the keypoint coordinates with a standard deviation of 2. This training mechanism allows the network to learn pose representations at different resolutions, improving robustness to scale variations. The heatmap loss ensures accurate keypoint localisation at each resolution scale (1/4 and 1/2).

1. **Convolutional Attention Block Module (CBAM)**

The Convolutional Block Attention Module^20^ is an attention mechanism designed to enhance feature representation in deep neural networks. CBAM has been widely used in pose detection^34–36^ CBAM to improve keypoint detection. It consists of two sequential attention modules performing channel attention and spatial attention, as depicted on Figure S7.a.


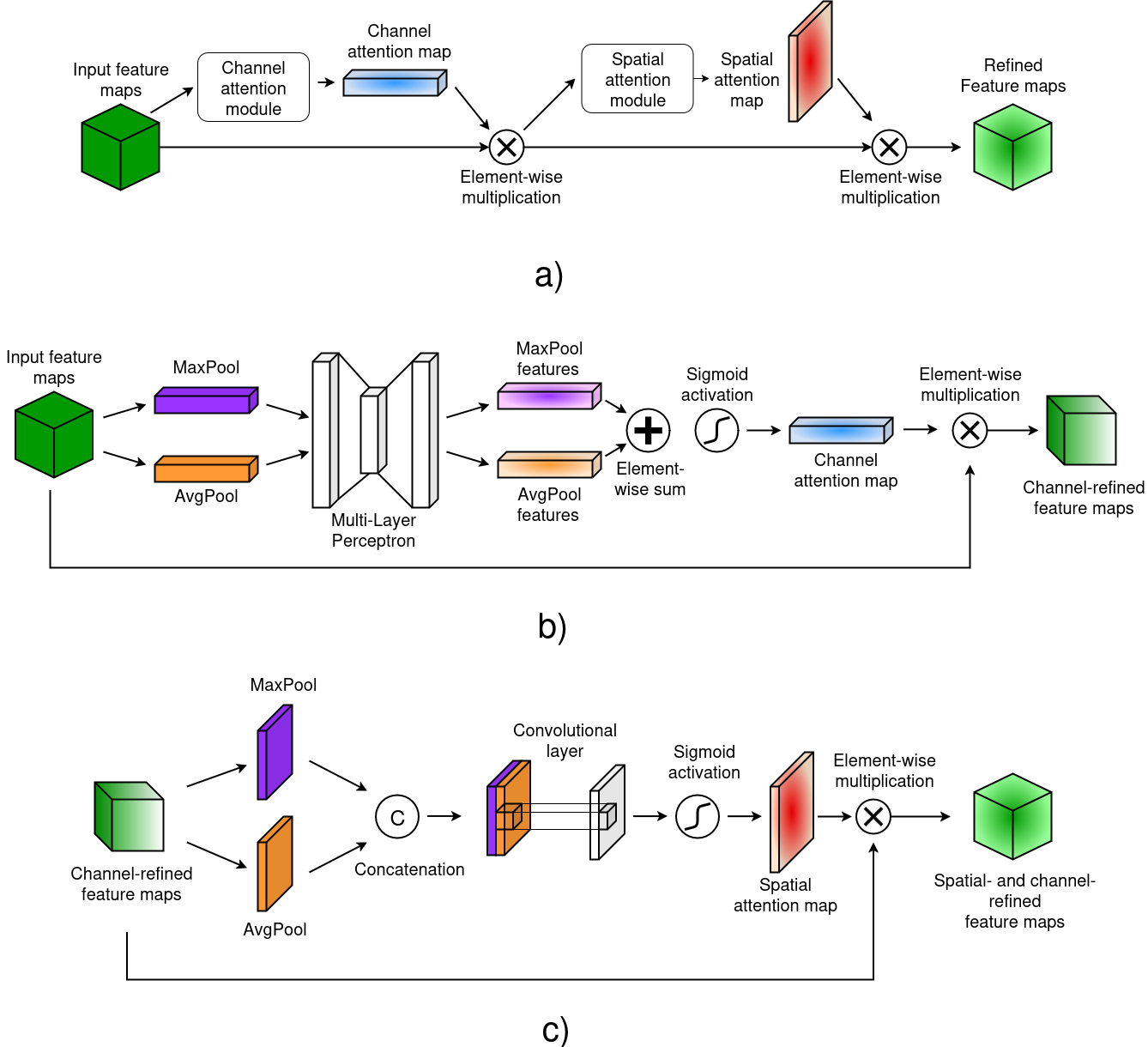


Figure S7 – Architecture of the convolutional block attention module (CBAM)^20^. a) Overview of CBAM that applies sequential channel and spatial attention to refine feature representation. b) Channel attention enhances important feature channels, while c) spatial attention highlights key spatial regions in the feature maps, improving the information retained in feature maps.

The channel attention module (Figure S7.b) refines feature maps by learning which feature map channels are the most relevant. It applies average pooling and max pooling throughout the spatial dimension of the input feature map, squeezing spatial dimensions and summarising each channel to a single value. Average pooling provides the average value of the spatial dimension, while max pooling retains the highest value of each channel. The pooled vectors are passed through a shared two-layer multi-layer perceptron with one hidden layer that reduces the channel dimension and learns the relationships between the average and max channel vectors. A dimension reduction ratio of 16 is conventionally selected, as it has empirically led to improved results^20^. The two vectors are summed element-wise and undergo a sigmoid activation that results in the channel attention map, which re-weights the channels‘ relative importance between 0 and 1. The input feature map is multiplied with the channel attention map to strengthen the most informative channels.

The spatial attention module (Figure S7.c) refines the feature maps by learning where important information is spatially located. It first performs by performing average and max pooling across the channels of the channel-refined feature map. Each pooling produces a spatial map that highlights the location of the average and peak values of each channel. This condenses channel information into spatial maps that translate the presence of features spatially. The resulting spatial maps are concatenated along the channel dimension and are processed by a convolutional layer with a 7x7 kernel to capture broader spatial dependencies. This outputs a single-channel attention map that reflects the relative importance of spatial features. A sigmoid activation is then applied to normalise the relative spatial relevance between 0 and 1. Finally, the channel-refined feature maps are multiplied by the spatial attention map to obtain a channel- spatial- refined feature map.

The resulting feature map is then passed down the network architecture in which CBAM is integrated.

1. **Loss function**

HigherHRNet is trained using heatmap loss on the 1/2 and 1/4 scale outputs. Heatmap loss allows supervision by predicting a probability distribution over keypoint locations rather than regressing they keypoints' coordinates. Instead of learning the $(x,y)$ coordinates of keypoints, the model outputs a heatmap for each keypoint. The intensity at each pixel location represents the likelihood of that pixel containing the keypoint. Given an image, the ground truth heatmap $H_{k}^{*}$ for a keypoint $k$ is modeled as a 2D Gaussian distribution centered at the keypoint's true location $\left( x_{k}^{*},y_{k}^{*} \right)$:

$$H_{k}^{*}=\exp\left( -\frac{\left( x-x_{k}^{*} \right)^{2}+\left( y-y_{k}^{*} \right)^{2}}{2\sigma_{k}^{2}} \right)$$

where $\sigma_{k}$ is a keypoint-specific standard deviation of the Gaussian distribution. $\sigma_{k}=1$ for smaller keypoints such as elbows and wrists, and $\sigma_{k}=2$ for larger keypoints such as head, shoulders and hips. These values follow the HigherHRNet implementation and encourage finer localisation for smaller keypoints while accounting for the larger spatial extend of larger ones. The predicted heatmap $H_{k}$ is then compared to the ground truth heatmap $H_{k}^{*}$ using a pixel-wise Mean Squared Error (MSE) loss:

$$\mathcal{L}_{heatmap}=\frac{1}{K}\sum_{i=1}^{K} \sum_{x,y} \left( H_{i}\left( x,y \right)-H_{i}^{*}\left( x,y \right) \right)^{2}$$

where $K$ is the total number of keypoints. The total heatmap loss for both scales is given by the heatmap loss at the 128x128 and 256x256 outputs:

$$\mathcal{L =}\mathcal{L}_{128x128}+\mathcal{L}_{256x256}$$

1. **Object Keypoint Similarity (OKS)**

Object Keypoint Similarity (OKS) is an evaluation metric used in body pose estimation to assess the accuracy of human pose estimation models. OKS was introduced by the authors of the Microsoft COCO dataset^29^. It serves as an analogue to the Intersection over Union (IoU) used in object detection but is specifically designed for keypoint-based annotations. OKS measures the similarity between predicted and ground-truth keypoints while accounting for variations in scale, occlusion, and keypoint importance. OKS is given by

$$OKS=\frac{\sum_{i=0}^{K} \exp\left( -\frac{d_{i}^{2}}{2s^{2}k_{i}^{2}} \right)\delta\left( v_{i}>0 \right)}{\sum_{i=0}^{K} \delta\left( v_{i}>0 \right)}$$

where $K$ is the set of predicted keypoints in a given human body instance, $d$ is the euclidian distance between the prediction and the ground-truth locations of the keypoint $i$, $s$ is the scale of the object, typically computed as the square root of its bounding box area, $k_{i}$ is a keypoint-specific constant that reflects the sensitivity to localisation errors, with smaller values assigned to keypoints that require higher precision (e.g., eyes, nose) and larger values to keypoints that allow more flexibility (e.g., hips, shoulders), $v_{i}$ is the visibility flag of keypoint $i$, where $v_{i}\in\left[ 0,1,2 \right]$ if the keypoint is unlabeled, labeled but not visible and visible respectively. $\delta\left( v_{i}>0 \right)$ is an impulse function that ensures only labelled keypoints contribute to the OKS.

OKS provides a similarity measure across individuals of different sizes and scales. It penalises large localisation errors more severely for small keypoints (such as facial landmarks) than for larger joints (like hips and shoulders), reflecting their relative importance in pose estimation.

**OKS-based metrics**

In human pose estimation, Object Keypoint Similarity (OKS) serves as the foundation for evaluation metrics, including Average Precision (AP) and Average Recall (AR). Precision is the proportion of true positive predictions among all positive predictions made by the model such as

$$Precision\boldsymbol{=}\frac{\text{True Positives}}{\text{True Positives}+\text{False Positives}}$$

Recall is the proportion of true positive predictions among all actual positive instances such as

$$Recall=\frac{\text{True Positives}}{\text{True Positives}+\text{False Negatives}}$$

These metrics assess a model’s ability to accurately predict keypoints by comparing them to ground-truth annotations across multiple OKS thresholds. Unlike traditional IoU-based measures, OKS accounts for scale variations and keypoint localisation precision, making it more suitable for pose estimation. AP measures how well a model predicts keypoints across different OKS thresholds. The COCO evaluation protocol computes AP by averaging precision values at thresholds ranging from 0.50 to 0.95 in increments of 0.05, ensuring both coarse and fine-grained localisation accuracy. The AP metrics we used include:

- **AP@0.50:0.95:** The mean Average Precision (mAP) across all OKS thresholds with an increment of 0.05.
- **AP@0.50 and AP@0.75**: Precision at lenient (0.50) and strict (0.75) OKS thresholds.

A higher AP indicates that a model consistently produces accurate keypoint predictions across various levels of precision. AR focuses on how many ground-truth keypoints are correctly detected at varying OKS thresholds. It is computed over multiple scales:

- **AR@0.50:0.95**: The mean Average Recall (mAR) score across all OKS thresholds with an increment of 0.05.
- **AR@0.50 and AR@0.75:** Recall at lenient and strict OKS thresholds.

While OKS-based metrics are standard in pose estimation, their reliability can be affected by the high rate of occluded keypoints and by the size of the motor event dataset.

1. Zoran Zivkovic and Ferdinand van der Heijden. Efficient adaptive density estimation per image pixel for the task of background subtraction. *Pattern recognition letters*, 27(7):773–780, 2006. [↑](#footnote-ref-1)
